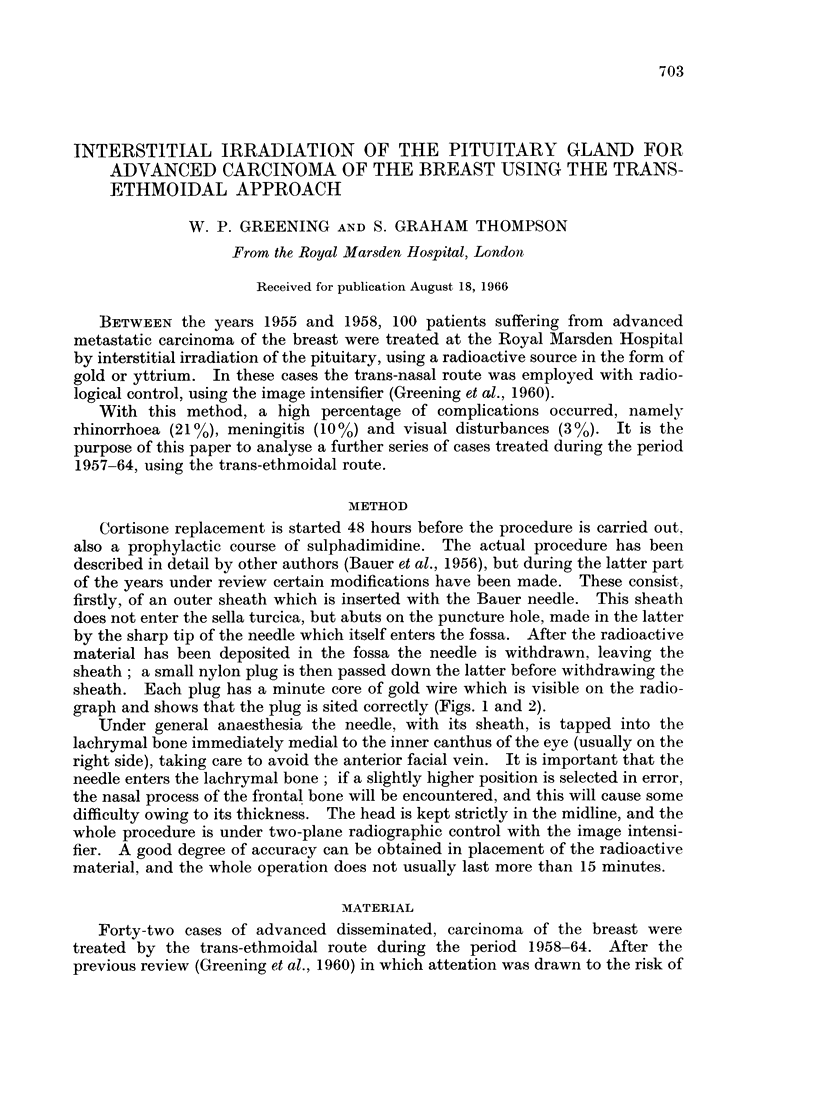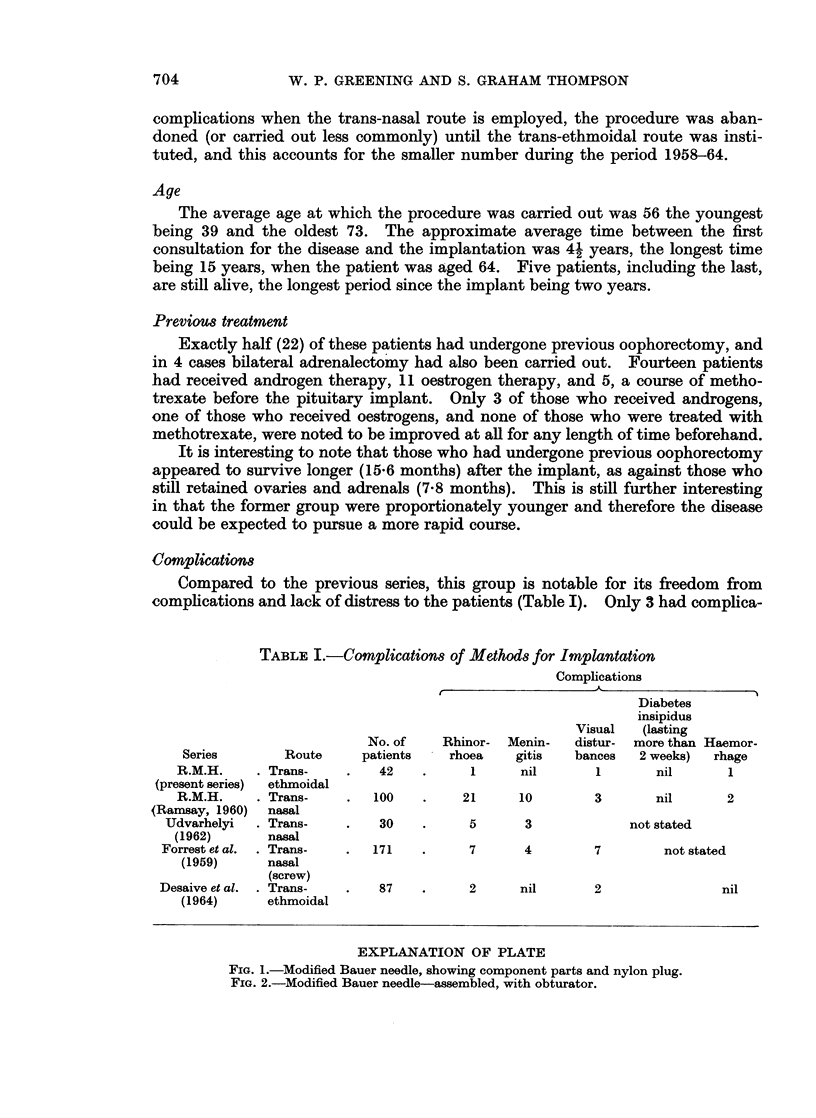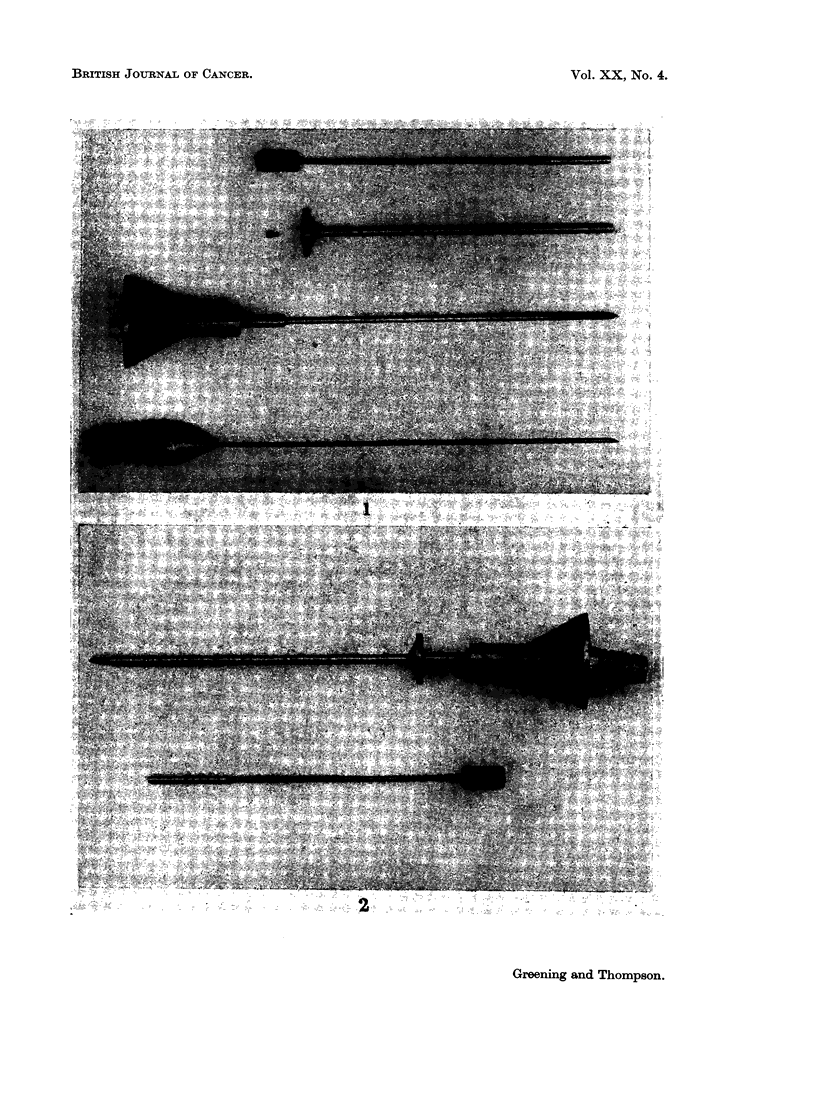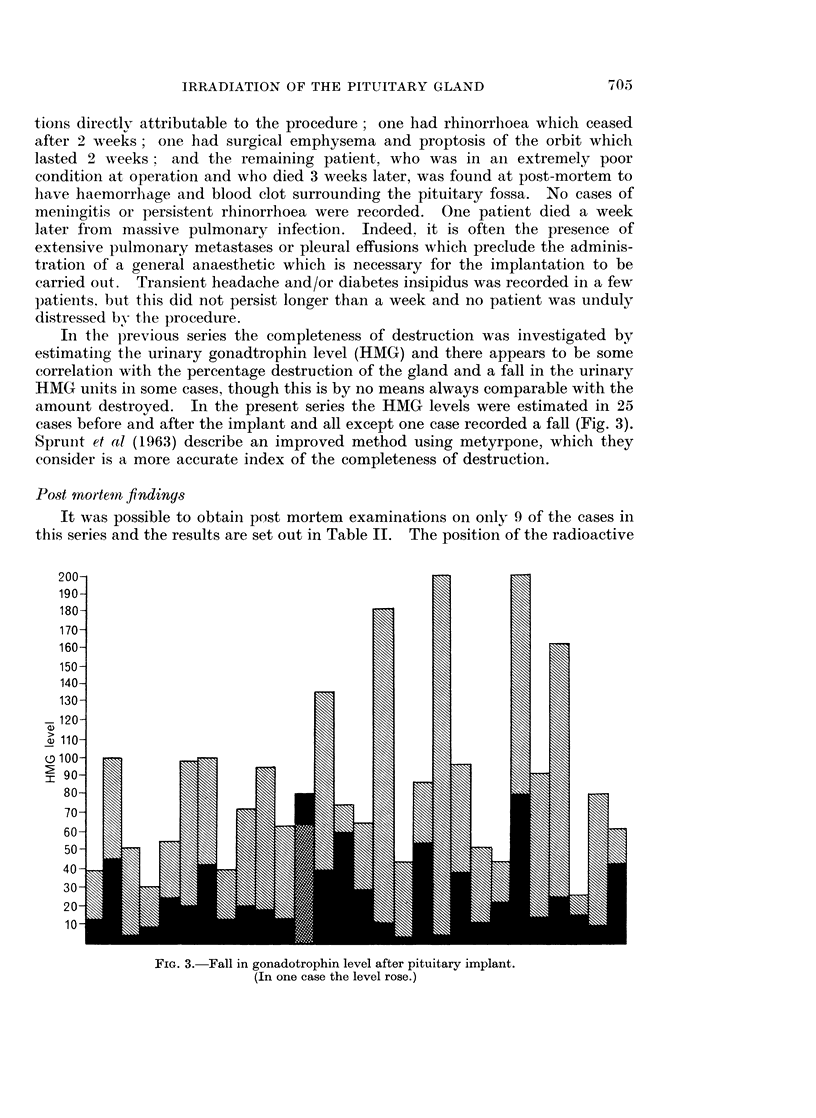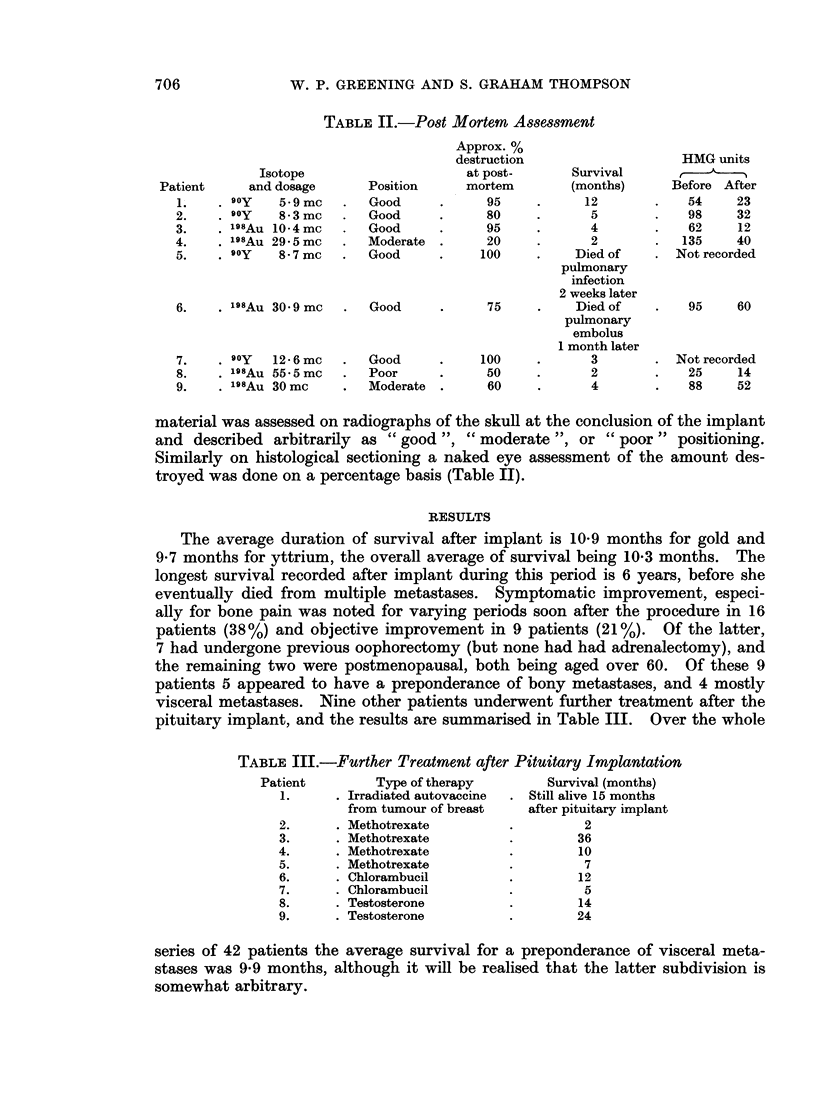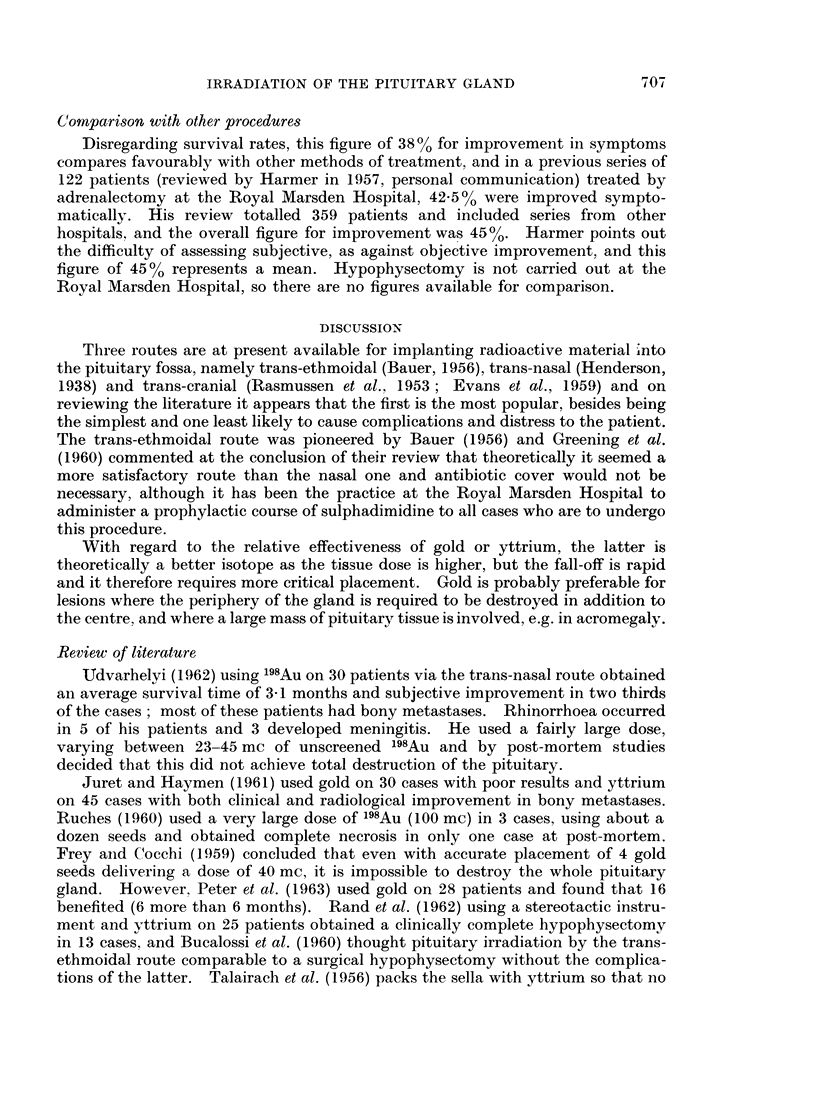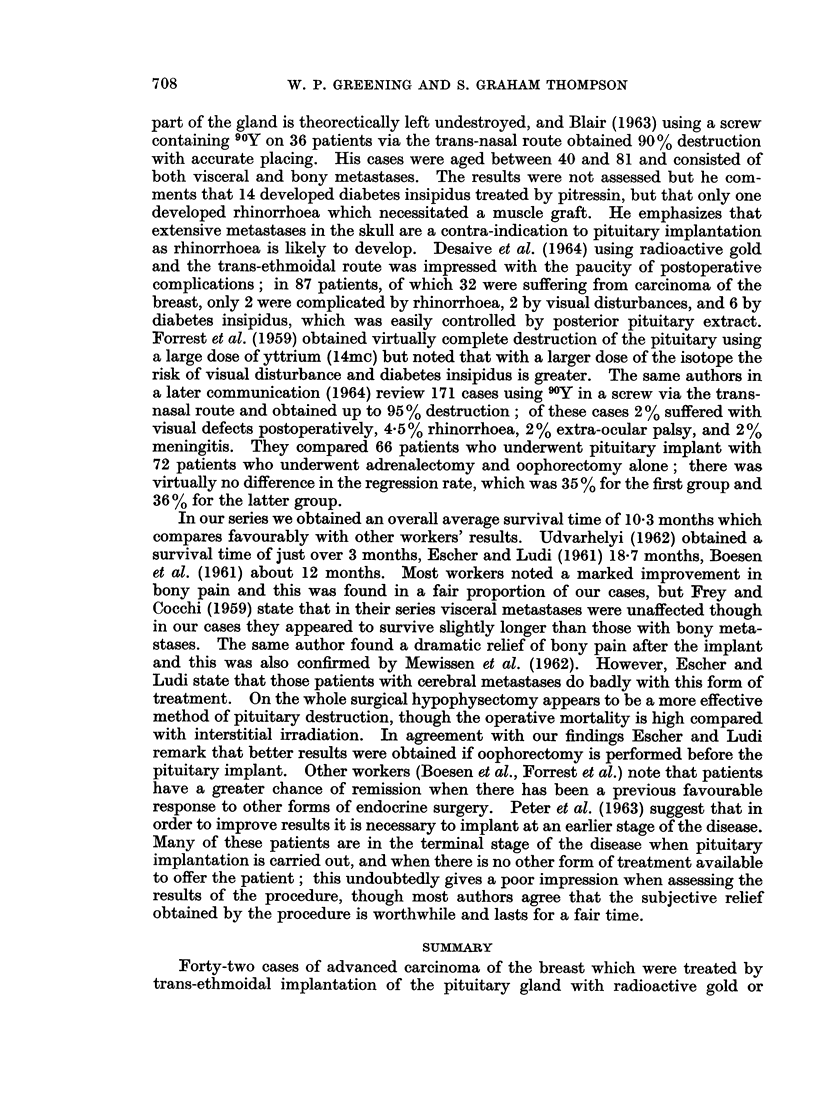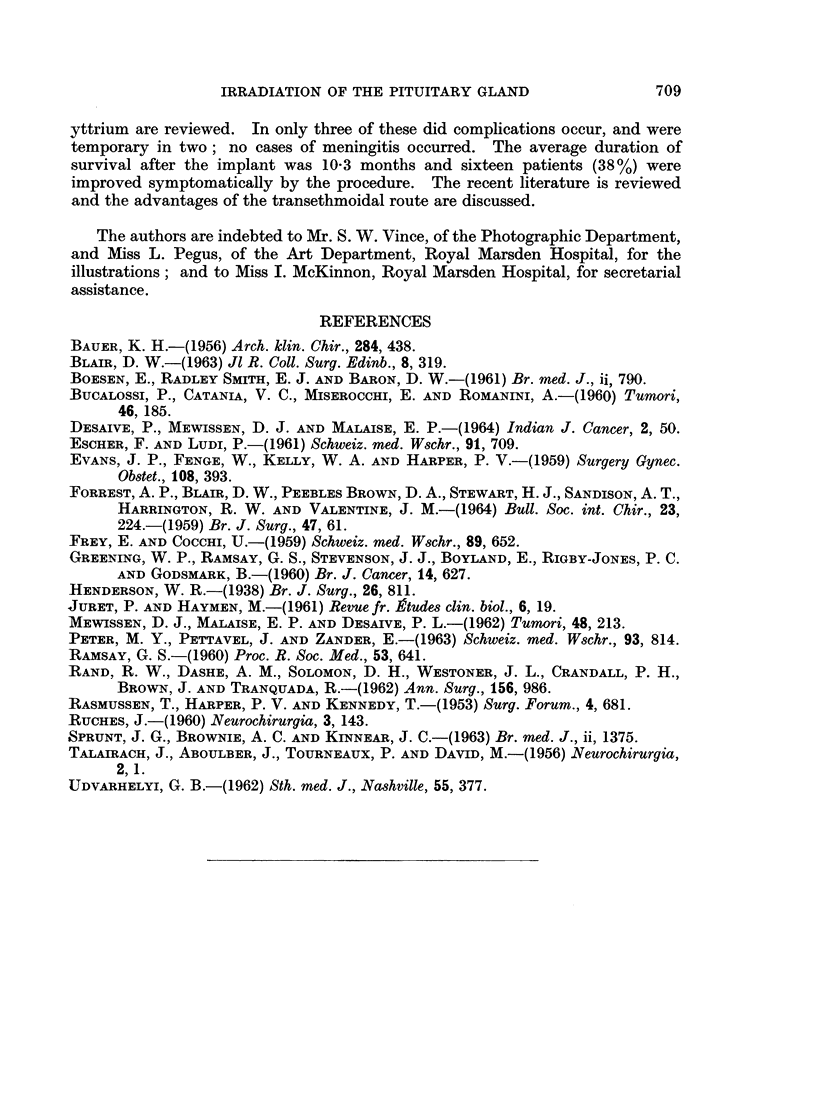# Interstitial irradiation of the pituitary gland for advanced carcinoma of the breast using the trans-ethmoidal approach.

**DOI:** 10.1038/bjc.1966.81

**Published:** 1966-12

**Authors:** W. P. Greening, S. G. Thompson

## Abstract

**Images:**


### INTERSTITIAL IRRADIATION OF THE PITUITARY GLAND FOR ADVANCED CARCINOMA OF THE BREAST USING THE TRANS-ETHMOIDAL APPROACH

W. P. GREENING AND S. GRAHAM THOMPSON

*From the Royal Marsden Hospital, London*

Received for publication August 18, 1966

BETWEEN the years 1955 and 1958, 100 patients suffering from advanced metastatic carcinoma of the breast were treated at the Royal Marsden Hospital by interstitial irradiation of the pituitary, using a radioactive source in the form of gold or yttrium. In these cases the trans-nasal route was employed with radiological control, using the image intensifier (Greening *et al.*, 1960).

With this method, a high percentage of complications occurred, namely rhinorrhoea (21%), meningitis (10%) and visual disturbances (3%). It is the purpose of this paper to analyse a further series of cases treated during the period 1957–64, using the trans-ethmoidal route.

### METHOD

Cortisone replacement is started 48 hours before the procedure is carried out, also a prophylactic course of sulphadimidine. The actual procedure has been described in detail by other authors (Bauer *et al.*, 1956), but during the latter part of the years under review certain modifications have been made. These consist, firstly, of an outer sheath which is inserted with the Bauer needle. This sheath does not enter the sella turcica, but abuts on the puncture hole, made in the latter by the sharp tip of the needle which itself enters the fossa. After the radioactive material has been deposited in the fossa the needle is withdrawn, leaving the sheath ; a small nylon plug is then passed down the latter before withdrawing the sheath. Each plug has a minute core of gold wire which is visible on the radiograph and shows that the plug is sited correctly (Figs. 1 and 2).

Under general anaesthesia the needle, with its sheath, is tapped into the lachrymal bone immediately medial to the inner canthus of the eye (usually on the right side), taking care to avoid the anterior facial vein. It is important that the needle enters the lachrymal bone ; if a slightly higher position is selected in error, the nasal process of the frontal bone will be encountered, and this will cause some difficulty owing to its thickness. The head is kept strictly in the midline, and the whole procedure is under two-plane radiographic control with the image intensifier. A good degree of accuracy can be obtained in placement of the radioactive material, and the whole operation does not usually last more than 15 minutes.

### MATERIAL

Forty-two cases of advanced disseminated, carcinoma of the breast were treated by the trans-ethmoidal route during the period 1958–64. After the previous review (Greening *et al.*, 1960) in which attention was drawn to the risk of complications when the trans-nasal route is employed, the procedure was abandoned (or carried out less commonly) until the trans-ethmoidal route was instituted, and this accounts for the smaller number during the period 1958–64.

### *Age*

The average age at which the procedure was carried out was 56 the youngest being 39 and the oldest 73. The approximate average time between the first consultation for the disease and the implantation was 4½ years, the longest time being 15 years, when the patient was aged 64. Five patients, including the last, are still alive, the longest period since the implant being two years.

### *Previous treatment*

Exactly half (22) of these patients had undergone previous oophorectomy, and in 4 cases bilateral adrenalectomy had also been carried out. Fourteen patients had received androgen therapy, 11 oestrogen therapy, and 5, a course of methotrexate before the pituitary implant. Only 3 of those who received androgens, one of those who received oestrogens, and none of those who were treated with methotrexate, were noted to be improved at all for any length of time beforehand.

It is interesting to note that those who had undergone previous oophorectomy appeared to survive longer (15·6 months) after the implant, as against those who still retained ovaries and adrenals (7·8 months). This is still further interesting in that the former group were proportionately younger and therefore the disease could be expected to pursue a more rapid course.

### *Complications*

Compared to the previous series, this group is notable for its freedom from complications and lack of distress to the patients (Table I). Only 3 had complica-

TABLE I.—*Complications of Methods for Implantation*

| | | | Complications | | | | |
|---|---|---|---|---|---|---|---|
| Series | Route | No. of patients | Rhinorrhoea | Meningitis | Visual disturbances | Diabetes insipidus (lasting more than 2 weeks) | Haemorrhage |
| R.M.H. (present series) | Trans-ethmoidal | 42 | 1 | nil | 1 | nil | 1 |
| R.M.H. (Ramsay, 1960) | Trans-nasal | 100 | 21 | 10 | 3 | nil | 2 |
| Udvarhelyi (1962) | Trans-nasal | 30 | 5 | 3 | | not stated | |
| Forrest *et al.* (1959) | Trans-nasal (screw) | 171 | 7 | 4 | 7 | not stated | |
| Desaive *et al.* (1964) | Trans-ethmoidal | 87 | 2 | nil | 2 | | nil |

EXPLANATION OF PLATE

FIG. 1.—Modified Bauer needle, showing component parts and nylon plug.
FIG. 2.—Modified Bauer needle—assembled, with obturator.

1

2

Greening and Thompson.

tions directly attributable to the procedure ; one had rhinorrhoea which ceased after 2 weeks ; one had surgical emphysema and proptosis of the orbit which lasted 2 weeks ; and the remaining patient, who was in an extremely poor condition at operation and who died 3 weeks later, was found at post-mortem to have haemorrhage and blood clot surrounding the pituitary fossa. No cases of meningitis or persistent rhinorrhoea were recorded. One patient died a week later from massive pulmonary infection. Indeed, it is often the presence of extensive pulmonary metastases or pleural effusions which preclude the administration of a general anaesthetic which is necessary for the implantation to be carried out. Transient headache and/or diabetes insipidus was recorded in a few patients, but this did not persist longer than a week and no patient was unduly distressed by the procedure.

In the previous series the completeness of destruction was investigated by estimating the urinary gonadtrophin level (HMG) and there appears to be some correlation with the percentage destruction of the gland and a fall in the urinary HMG units in some cases, though this is by no means always comparable with the amount destroyed. In the present series the HMG levels were estimated in 25 cases before and after the implant and all except one case recorded a fall (Fig. 3). Sprunt *et al* (1963) describe an improved method using metyrpone, which they consider is a more accurate index of the completeness of destruction.

*Post mortem findings*

It was possible to obtain post mortem examinations on only 9 of the cases in this series and the results are set out in Table II. The position of the radioactive

FIG. 3.—Fall in gonadotrophin level after pituitary implant.
(In one case the level rose.)

TABLE II.—*Post Mortem Assessment*

| Patient | Isotope and dosage | Position | Approx. % destruction at post-mortem | Survival (months) | HMG units Before | HMG units After |
|---|---|---|---|---|---|---|
| 1. | $^{90}$Y 5·9 mc | Good | 95 | 12 | 54 | 23 |
| 2. | $^{90}$Y 8·3 mc | Good | 80 | 5 | 98 | 32 |
| 3. | $^{198}$Au 10·4 mc | Good | 95 | 4 | 62 | 12 |
| 4. | $^{198}$Au 29·5 mc | Moderate | 20 | 2 | 135 | 40 |
| 5. | $^{90}$Y 8·7 mc | Good | 100 | Died of pulmonary infection 2 weeks later | Not recorded | |
| 6. | $^{198}$Au 30·9 mc | Good | 75 | Died of pulmonary embolus 1 month later | 95 | 60 |
| 7. | $^{90}$Y 12·6 mc | Good | 100 | 3 | Not recorded | |
| 8. | $^{198}$Au 55·5 mc | Poor | 50 | 2 | 25 | 14 |
| 9. | $^{198}$Au 30 mc | Moderate | 60 | 4 | 88 | 52 |

material was assessed on radiographs of the skull at the conclusion of the implant and described arbitrarily as "good", "moderate", or "poor" positioning. Similarly on histological sectioning a naked eye assessment of the amount destroyed was done on a percentage basis (Table II).

### RESULTS

The average duration of survival after implant is 10·9 months for gold and 9·7 months for yttrium, the overall average of survival being 10·3 months. The longest survival recorded after implant during this period is 6 years, before she eventually died from multiple metastases. Symptomatic improvement, especially for bone pain was noted for varying periods soon after the procedure in 16 patients (38%) and objective improvement in 9 patients (21%). Of the latter, 7 had undergone previous oophorectomy (but none had had adrenalectomy), and the remaining two were postmenopausal, both being aged over 60. Of these 9 patients 5 appeared to have a preponderance of bony metastases, and 4 mostly visceral metastases. Nine other patients underwent further treatment after the pituitary implant, and the results are summarised in Table III. Over the whole

TABLE III.—*Further Treatment after Pituitary Implantation*

| Patient | Type of therapy | Survival (months) |
|---|---|---|
| 1. | Irradiated autovaccine from tumour of breast | Still alive 15 months after pituitary implant |
| 2. | Methotrexate | 2 |
| 3. | Methotrexate | 36 |
| 4. | Methotrexate | 10 |
| 5. | Methotrexate | 7 |
| 6. | Chlorambucil | 12 |
| 7. | Chlorambucil | 5 |
| 8. | Testosterone | 14 |
| 9. | Testosterone | 24 |

series of 42 patients the average survival for a preponderance of visceral metastases was 9·9 months, although it will be realised that the latter subdivision is somewhat arbitrary.

*Comparison with other procedures*

Disregarding survival rates, this figure of 38% for improvement in symptoms compares favourably with other methods of treatment, and in a previous series of 122 patients (reviewed by Harmer in 1957, personal communication) treated by adrenalectomy at the Royal Marsden Hospital, 42·5% were improved symptomatically. His review totalled 359 patients and included series from other hospitals, and the overall figure for improvement was 45%. Harmer points out the difficulty of assessing subjective, as against objective improvement, and this figure of 45% represents a mean. Hypophysectomy is not carried out at the Royal Marsden Hospital, so there are no figures available for comparison.

### DISCUSSION

Three routes are at present available for implanting radioactive material into the pituitary fossa, namely trans-ethmoidal (Bauer, 1956), trans-nasal (Henderson, 1938) and trans-cranial (Rasmussen *et al.*, 1953 ; Evans *et al.*, 1959) and on reviewing the literature it appears that the first is the most popular, besides being the simplest and one least likely to cause complications and distress to the patient. The trans-ethmoidal route was pioneered by Bauer (1956) and Greening *et al.* (1960) commented at the conclusion of their review that theoretically it seemed a more satisfactory route than the nasal one and antibiotic cover would not be necessary, although it has been the practice at the Royal Marsden Hospital to administer a prophylactic course of sulphadimidine to all cases who are to undergo this procedure.

With regard to the relative effectiveness of gold or yttrium, the latter is theoretically a better isotope as the tissue dose is higher, but the fall-off is rapid and it therefore requires more critical placement. Gold is probably preferable for lesions where the periphery of the gland is required to be destroyed in addition to the centre, and where a large mass of pituitary tissue is involved, e.g. in acromegaly.

*Review of literature*

Udvarhelyi (1962) using $^{198}$Au on 30 patients via the trans-nasal route obtained an average survival time of 3·1 months and subjective improvement in two thirds of the cases ; most of these patients had bony metastases. Rhinorrhoea occurred in 5 of his patients and 3 developed meningitis. He used a fairly large dose, varying between 23–45 mc of unscreened $^{198}$Au and by post-mortem studies decided that this did not achieve total destruction of the pituitary.

Juret and Haymen (1961) used gold on 30 cases with poor results and yttrium on 45 cases with both clinical and radiological improvement in bony metastases. Ruches (1960) used a very large dose of $^{198}$Au (100 mc) in 3 cases, using about a dozen seeds and obtained complete necrosis in only one case at post-mortem. Frey and Cocchi (1959) concluded that even with accurate placement of 4 gold seeds delivering a dose of 40 mc, it is impossible to destroy the whole pituitary gland. However, Peter *et al.* (1963) used gold on 28 patients and found that 16 benefited (6 more than 6 months). Rand *et al.* (1962) using a stereotactic instrument and yttrium on 25 patients obtained a clinically complete hypophysectomy in 13 cases, and Bucalossi *et al.* (1960) thought pituitary irradiation by the trans-ethmoidal route comparable to a surgical hypophysectomy without the complications of the latter. Talairach *et al.* (1956) packs the sella with yttrium so that no part of the gland is theorectically left undestroyed, and Blair (1963) using a screw containing $^{90}Y$ on 36 patients via the trans-nasal route obtained 90% destruction with accurate placing. His cases were aged between 40 and 81 and consisted of both visceral and bony metastases. The results were not assessed but he comments that 14 developed diabetes insipidus treated by pitressin, but that only one developed rhinorrhoea which necessitated a muscle graft. He emphasizes that extensive metastases in the skull are a contra-indication to pituitary implantation as rhinorrhoea is likely to develop. Desaive *et al.* (1964) using radioactive gold and the trans-ethmoidal route was impressed with the paucity of postoperative complications ; in 87 patients, of which 32 were suffering from carcinoma of the breast, only 2 were complicated by rhinorrhoea, 2 by visual disturbances, and 6 by diabetes insipidus, which was easily controlled by posterior pituitary extract. Forrest *et al.* (1959) obtained virtually complete destruction of the pituitary using a large dose of yttrium (14mc) but noted that with a larger dose of the isotope the risk of visual disturbance and diabetes insipidus is greater. The same authors in a later communication (1964) review 171 cases using $^{90}Y$ in a screw via the trans-nasal route and obtained up to 95% destruction ; of these cases 2% suffered with visual defects postoperatively, 4·5% rhinorrhoea, 2% extra-ocular palsy, and 2% meningitis. They compared 66 patients who underwent pituitary implant with 72 patients who underwent adrenalectomy and oophorectomy alone ; there was virtually no difference in the regression rate, which was 35% for the first group and 36% for the latter group.

In our series we obtained an overall average survival time of 10·3 months which compares favourably with other workers' results. Udvarhelyi (1962) obtained a survival time of just over 3 months, Escher and Ludi (1961) 18·7 months, Boesen *et al.* (1961) about 12 months. Most workers noted a marked improvement in bony pain and this was found in a fair proportion of our cases, but Frey and Cocchi (1959) state that in their series visceral metastases were unaffected though in our cases they appeared to survive slightly longer than those with bony metastases. The same author found a dramatic relief of bony pain after the implant and this was also confirmed by Mewissen *et al.* (1962). However, Escher and Ludi state that those patients with cerebral metastases do badly with this form of treatment. On the whole surgical hypophysectomy appears to be a more effective method of pituitary destruction, though the operative mortality is high compared with interstitial irradiation. In agreement with our findings Escher and Ludi remark that better results were obtained if oophorectomy is performed before the pituitary implant. Other workers (Boesen *et al.*, Forrest *et al.*) note that patients have a greater chance of remission when there has been a previous favourable response to other forms of endocrine surgery. Peter *et al.* (1963) suggest that in order to improve results it is necessary to implant at an earlier stage of the disease. Many of these patients are in the terminal stage of the disease when pituitary implantation is carried out, and when there is no other form of treatment available to offer the patient ; this undoubtedly gives a poor impression when assessing the results of the procedure, though most authors agree that the subjective relief obtained by the procedure is worthwhile and lasts for a fair time.

### SUMMARY

Forty-two cases of advanced carcinoma of the breast which were treated by trans-ethmoidal implantation of the pituitary gland with radioactive gold or yttrium are reviewed. In only three of these did complications occur, and were temporary in two; no cases of meningitis occurred. The average duration of survival after the implant was 10·3 months and sixteen patients (38%) were improved symptomatically by the procedure. The recent literature is reviewed and the advantages of the transethmoidal route are discussed.

The authors are indebted to Mr. S. W. Vince, of the Photographic Department, and Miss L. Pegus, of the Art Department, Royal Marsden Hospital, for the illustrations; and to Miss I. McKinnon, Royal Marsden Hospital, for secretarial assistance.

### REFERENCES

BAUER, K. H.—(1956) *Arch. klin. Chir.*, **284**, 438.

BLAIR, D. W.—(1963) *Jl R. Coll. Surg. Edinb.*, **8**, 319.

BOESEN, E., RADLEY SMITH, E. J. AND BARON, D. W.—(1961) *Br. med. J.*, ii, 790.

BUCALOSSI, P., CATANIA, V. C., MISEROCCHI, E. AND ROMANINI, A.—(1960) *Tumori*, **46**, 185.

DESAIVE, P., MEWISSEN, D. J. AND MALAISE, E. P.—(1964) *Indian J. Cancer*, **2**, 50.

ESCHER, F. AND LUDI, P.—(1961) *Schweiz. med. Wschr.*, **91**, 709.

EVANS, J. P., FENGE, W., KELLY, W. A. AND HARPER, P. V.—(1959) *Surgery Gynec. Obstet.*, **108**, 393.

FORREST, A. P., BLAIR, D. W., PEEBLES BROWN, D. A., STEWART, H. J., SANDISON, A. T., HARRINGTON, R. W. AND VALENTINE, J. M.—(1964) *Bull. Soc. int. Chir.*, **23**, 224.—(1959) *Br. J. Surg.*, **47**, 61.

FREY, E. AND COCCHI, U.—(1959) *Schweiz. med. Wschr.*, **89**, 652.

GREENING, W. P., RAMSAY, G. S., STEVENSON, J. J., BOYLAND, E., RIGBY-JONES, P. C. AND GODSMARK, B.—(1960) *Br. J. Cancer*, **14**, 627.

HENDERSON, W. R.—(1938) *Br. J. Surg.*, **26**, 811.

JURET, P. AND HAYMEN, M.—(1961) *Revue fr. Études clin. biol.*, **6**, 19.

MEWISSEN, D. J., MALAISE, E. P. AND DESAIVE, P. L.—(1962) *Tumori*, **48**, 213.

PETER, M. Y., PETTAVEL, J. AND ZANDER, E.—(1963) *Schweiz. med. Wschr.*, **93**, 814.

RAMSAY, G. S.—(1960) *Proc. R. Soc. Med.*, **53**, 641.

RAND, R. W., DASHE, A. M., SOLOMON, D. H., WESTONER, J. L., CRANDALL, P. H., BROWN, J. AND TRANQUADA, R.—(1962) *Ann. Surg.*, **156**, 986.

RASMUSSEN, T., HARPER, P. V. AND KENNEDY, T.—(1953) *Surg. Forum.*, **4**, 681.

RUCHES, J.—(1960) *Neurochirurgia*, **3**, 143.

SPRUNT, J. G., BROWNIE, A. C. AND KINNEAR, J. C.—(1963) *Br. med. J.*, ii, 1375.

TALAIRACH, J., ABOULBER, J., TOURNEAUX, P. AND DAVID, M.—(1956) *Neurochirurgia*, **2**, 1.

UDVARHELYI, G. B.—(1962) *Sth. med. J., Nashville*, **55**, 377.